# Comparison of LAMP and PCR for molecular mass screening of sand flies for *Leishmania martiniquensis* infection

**DOI:** 10.1590/0074-02760160254

**Published:** 2017-02

**Authors:** Saruda Tiwananthagorn, Hirotomo Kato, Ranchana Yeewa, Amontip Muengpan, Raxsina Polseela, Saovanee Leelayoova

**Affiliations:** 1 Chiang Mai University, Faculty of Veterinary Medicine, Department of Veterinary Biosciences and Veterinary Public Health, Muang, Chiang Mai, Thailand; 2 Jichi Medical University, Department of Infection and Immunity, Division of Medical Zoology, Tochigi, Japan; 3 Naresuan University, Faculty of Medical Science, Department of Microbiology and Parasitology, Phitsanulok, Thailand; 4 Phramongkutklao College of Medicine, Department of Parasitology, Bangkok, Thailand

**Keywords:** Leishmania martiniquensis, PCR, minicircle kinetoplast DNA, Loop-mediated isothermal amplification, molecular screening, individual sand fly

## Abstract

**BACKGROUND:**

Leishmaniasis caused by *Leishmania martiniquensis* infection has been reported in human and domestic animals of Martinique Island, Germany, Switzerland, USA, Myanmar and Thailand. The peculiar clinical features of disseminated cutaneous and visceral forms co-existence render the urgent need of specific diagnostic tool to identify the natural sand fly vectors for effective prevention and control strategies. Loop-mediated isothermal amplification (LAMP) of *18S rRNA* gene as well as polymerase chain reaction (PCR) of *minicircle kinetoplast DNA* gene (PCR-mkDNA) have never been applied to detect *L. martiniquensis* and *L. siamensis* in sand fly vectors.

**OBJECTIVE:**

The present study was aimed to validate malachite green-LAMP (MG-LAMP) and PCR-mkDNA techniques to detect *L. martiniquensis* in sand fly vectors, compared with the conventional PCR of *internal transcribed spacer 1* (PCR-ITS1).

**METHODS:**

We compared the validity of LAMP of *18S rRNA* gene and PCR-mkDNA, to PCR-ITS1 in simulation model of *L. martiniquensis* infection in *Sergentomyia gemmea* sand flies. Attributable to the sensitivity and specificity, PCR-mkDNA was consecutively applied to detect *L. martiniquensis* in 380 female sand fly individuals captured in the newly identified affected region of Lamphun Province, Thailand.

**FINDINGS AND MAIN CONCLUSIONS:**

Results showed that PCR-mkDNA could detect at least one promastigote per sand fly, which was 10-time superior to LAMP and PCR-ITS1. In addition, PCR-mkDNA was more specific, able to differentiate *L. martiniquensis* from other viscerotropic *Leishmania* species, such as *L. siamensis*, *L. (L.) donovani*, and *L. (L.) infantum.* Consecutively, mass screening of *L. martiniquensis* in 380 female sand fly individuals by PCR-mkDNA was implemented in a new affected area of Thailand where a patient with leishmaniasis/HIV co-infection resides; however *Leishmania* DNA was undetected. In conclusion, PCR-mkDNA is a promising tool for molecular mass screening of *L. martiniquensis* infection in outbreak areas where several species of *Leishmania* and sand flies co-exist.

Leishmaniasis is a vector-borne protozoan disease caused by several species of the genus *Leishmania*. Main clinical manifestations include cutaneous leishmaniasis (CL), mucocutaneous leishmaniasis (MCL), and visceral leishmaniasis (VL), generally associated with the *Leishmania* species. *Leishmania* promastigote develop in the gut of female sand flies, and differentiate into intracellular amastigote forms in vertebrate hosts after transmission. The spread of disease depends on the distribution of the vectors and reservoir animal hosts. Autochthonous cutaneous and VL is now considered an emerging disease in Thailand (Leelayoova et al. 2013, Chiewchanvit et al. 2015). Characterisation of *Leishmania* isolates in Thailand is based on sequence analysis of the *internal transcribed spacer 1* (*ITS1*) and the large subunit of *RNA polymerase* II genes suggesting that two distinct species belonging to *L. enriettii* complex are the causative agents; *L. siamensis* and *L. martiniquensis* (Pothirat et al. 2014, Chiewchanvit et al. 2015). In contrast to *L. siamensis* that was isolated only in one patient from Trang Province (Leelayoova et al. 2013), *L. martiniquensis* is more dominant and has a wider geographical distribution, including France, Germany, Switzerland, USA, Myanmar and Thailand (Chiewchanvit et al. 2015).

*L. martiniquensis* was first isolated in Martinique, Caribbean, in 1995; its taxonomic position was established in 2002, and it was named as *Leishmania (Leishmania) martiniquensis*, Desbois, Pratlong and Dedet n. sp. in 2014 (Dedet et al. 1995, Noyes et al. 2002, Desbois et al. 2014). This species was also considered in southern Thailand and Myanmar causing various clinical manifestations from asymptomatic, CL alone, VL alone, and atypically disseminated cutaneous and visceral co-existing forms (Phumee et al. 2013, Chiewchanvit et al. 2015), especially in people with human immunodeficiency virus co-infection. Regarding to animal reservoirs and vectors, *Leishmania* DNA was identified in black rats (*Rattus rattus*) and in two species of sand flies; *Sergentomyia (Neophlebotomus) gemmea* and *S. barraudi* in southern Thailand (Kanjanopas et al. 2013, Chusri et al. 2014). Between 2011 and 2014, at least five cases of *L. martiniquensis* infection have occurred in northern Thailand including one case in Chiang Rai province (Phumee et al. 2013); one case in Chiang Mai province (Chiewchanvit et al. 2015) and three cases in Lamphun province (BVBD/MoPH 2013, Pothirat et al. 2014, Chiewchanvit et al. 2015). Due to the continually increasing number of cases of *L. martiniquensis* in Thailand, the development of a specific diagnostic tool to identify *Leishmania* infection in circulating sand flies in the affected areas is urgently needed.

Detecting and identifying *Leishmania* species in sand flies and animal reservoirs are important to predict the risk and transmission of the disease in outbreak and surrounding areas (Kato et al. 2007, 2010). Molecular techniques, such as polymerase chain reaction (PCR) and PCR-restriction fragment length polymorphism (PCR-RFLP) have been applied to detect and identify *Leishmania* species in reservoir hosts and sand fly vectors with high sensitivity and specificity (Kato et al. 2007, 2010). Due to various limitations in the microscopic detection of *Leishmania* in sand flies, a molecular mass screening method for *Leishmania* infection of sand fly individuals has been established (Kato et al. 2007, 2010). This method is a powerful tool for research confirmed on prevalent sand fly species and vector-host-parasite inter-relationships (Kato et al. 2007, 2010, Tiwananthagorn et al. 2012). PCR targeting various genes, such as *ITS1*, *small subunit 18S ribosomal RNA* (*18S rRNA*), *minicircle kinetoplast DNA* (*mkDNA*), mitochondrial *cytochrome* b (*cyt* b), have been used to identify *Leishmania* infection in sand flies (Kato et al. 2010, Kanjanopas et al. 2013, Chusri et al. 2014), human patients and animal reservoirs (Leelayoova et al. 2013, Chusri et al. 2014, Hitakarun et al. 2014, Chiewchanvit et al. 2015). PCR targeting the *mkDNA* gene (PCR-mkDNA) has high sensitivity even when only one *Leishmania* parasite exists in a sample (Kato et al. 2007). PCR targeting the *ITS1* gene (PCR-ITS1) showed high sensitivity to detect *L. siamensis* as low as 0.05 promastigotes/µL (Hitakarun et al. 2014) and is the classical technique to detect *L. siamensis* and *L. martiniquensis* in sand fly vectors (Kanjanopas et al. 2013, Chusri et al. 2014).

A colorimetric malachite green based Loop-mediated isothermal amplification (MG-LAMP) assay targeting the *18S rRNA* gene has been developed for the robustness and superior sensitivity for mass screening of *L. mexicana* and *L. major* infection in sand flies, with a detection sensitivity of 0.01 parasite (Nzelu et al. 2014). Recently, the LAMP assay has been developed for simple detection of *L. siamensis* in clinical samples with the low detection limit as 10^3^ parasites/mL whole blood or 2.5 parasites/tube (Sriworarat et al. 2015). However, LAMP as well as PCR-mkDNA have never been applied to detect *L. martiniquensis* and *L. siamensis* in sand fly vectors. The present study, therefore, was aimed to validate MG-LAMP and PCR-mkDNA techniques to detect *L. martiniquensis* in sand fly vectors, compared with the conventional PCR-ITS1. Attributable to the sensitivity and specificity, PCR-mkDNA was consecutively applied to detect *L. martiniquensis* in 380 female sand fly individuals captured in the newly identified affected region of Lamphun Province.

## MATERIALS AND METHODS

*Parasites* - Promastigotes of *L. martiniquensis* (MHOM/TH/2011/PG) were harvested from axenic culture in Schneider’s *Drosophila* medium with L-glutamate (Sigma-Aldrich, USA), supplemented with 20% fetal bovine serum (Merck Millipore, Germany), 100 U/mL penicillin, 100 μg/mL streptomycin, 50 µg/mL gentamicin at 25ºC.

*Sand fly collection and taxonomic identification* - Sand flies were collected during October 2014 to May 2015 from a new affected area of Tha Mae Lop Subdistrict, Mae Tha District, Lamphun Province (Supplementary data), where a patient with autochthonous disseminated leishmaniasis caused by *L. martiniquensis* resides (Chiewchanvit et al. 2015). The sites were the patient’s house and the surrounding areas at a radius of 200 m. Collections using CDC light traps were conducted for 12 h between 6:00 pm and 6:00 am both indoors (living room, kitchen), and outdoors (animal shed, crafting studio, animal burrow), bamboo plantation, as well as Doi Khurea mountain (altitude 480 m), where the patient has been working as a lumberjack. All sand flies were stored individually in absolute ethanol and kept at -20ºC until further examination.

Each unfed and blood-fed female sand fly was dissected using sterile techniques under a stereomicroscope. The head and last three abdominal segments of each sand fly were mounted on a microscopic slide in Hoyer’s medium. Taxonomic identification was conducted morphologically following Lewis keys (Lewis 1978), such as morphology of cibarium and spermatheca. The remnant parts of sand flies were stored in absolute ethanol individually and kept at -20ºC until DNA was extracted.

*DNA preparation* - For the preparation of parasite DNA, 10,000 promastigotes of *L. martiniquensis* were suspended in 50 μL of DNA extraction buffer (150 mM NaCl, 10 mM Tris-HCl [pH 8.0], 10 mM EDTA, and 0.1% sodium dodecyl sulfate) in the presence of proteinase K (200 μg/mL), and serially diluted 10-fold in the same buffer. The samples, without homogenisation, were then incubated at 56ºC for 12 h, heat inactivated at 95ºC for 5 min, and 25 μL distilled water was added. The DNA samples were stored at -20ºC for further use.

To extract DNA from sand flies, a mass extraction technique (Kato et al. 2007) was implemented with a minor modification. Briefly, the ethanol-fixed sand fly specimens were placed individually in each microcentrifuge tube and lysed in 50 μL DNA extraction buffer without homogenisation. The samples were then processed and stored, as mentioned above.

DNA samples of other *Leishmania* species used in this study were prepared from the following reference strains, including *L. siamensis* (MHOM/TH/2010/TR), *L. martiniquensis* (MHOM/TH/2013/LSCM3), *L. (L.) major* (MHOM/SU/1973/5ASKH), *L. (L.) amazonensis* (MHOM/BR/1973/M2269), *L. (Viannia) braziliensis* (MHOM/BR/1973/M2269), *L. (L.) infantum* (MCAN/TR/2000/EP55), and *L. (L.) donovani* (MHOM/SU/62/2S-25M-C2). In addition, DNA samples of the local stains of *Trypanosoma evansi*, *Leucocytozoon sabrazesi*, and *Plasmodium gallinaceum* were used for the specificity test in this study.

*LAMP and PCR assays* - MG-LAMP assay targeting the *Leishmania 18S rRNA* gene (Nzelu et al. 2014) was validated for its sensitivity and specificity for *L. martiniquensis* detection. Briefly, the reaction was conducted in 15 μL of a reaction mixture consisting of 1.6 μM of each inner primer (FIP and BIP), a 0.4 μM of each outer primer (F3 and B3), 1x reaction mix (Eiken, Japan), 8 U *Bst* DNA polymerase (Eiken), 0.004% malachite green (MG) dye (dissolved in distilled water), and 1 μL of template DNA. The mixture was incubated at 64ºC for 60 min and then heated at 80ºC to terminate the reaction using MJ Research PTC-200 Thermal Cycler (Bio-Rad Laboratories, CA). At the end of incubation, the amplification of the target gene was confirmed based on direct visual inspection of the reaction tubes by the naked eye; a positive amplification showed as light blue, whereas in the absence of amplification, the reaction mixture became colorless. In addition, LAMP products were analysed on a 2.5% agarose gel electrophoresis.

PCR-mkDNA using primer L.MC-1S/ L.MC-1R (Kato et al. 2007) and PCR-ITS1 using primer L5.8S/ LITSR (El Tai et al. 2001) were conducted as previously described. Briefly, PCR was carried out in a volume of 20 μL using the primers (0.4 μM each), Ampdirect Plus (Shimadzu Biotech, Japan), and 0.5 U BioTaq™ HS DNA polymerase (Bioline, UK) with 1 μL of template DNA. After an initial denaturation at 95ºC for 10 min, PCR amplification was performed with 35 cycles of denaturation (95ºC, 1 min), annealing (55ºC, 45 s for PCR-mkDNA or 53ºC, 30 s for PCR-ITS1), and polymerisation (72ºC, 1 min) followed by a final extension at 72ºC for 10 min. The PCR products were analysed on a 1.5% agarose gel electrophoresis.

To identify sand fly species using molecular techniques, PCR and sequencing of the gene mitochondrial *cytochrome oxidase* subunit I (COI) of metazoan invertebrate (LCO1490/HCO2198) were performed, with the conditions described previously (Nzelu et al. 2015). All PCR products were purified using a QIAquick PCR purification kit (QIAGEN, Germany) and subsequently sent to Applied Biosystems DNA sequencing service (Thermo Fisher Scientific, Japan) for direct sequencing. The sequences were analysed by nucleotide BLAST program (National Center for Biotechnology Information, National Library of Medicine, Bethesda, USA). The sequences were aligned by Clustal W incorporated into MEGA (Molecular Evolutionary Genetics Analysis) version 6 (Tamura et al. 2013). The nucleotide compositions and sequence divergences within and between species were calculated using the distance model Kimura 2-Parameter. A neighbor-joining tree of Kimura 2-Parameter distances with bootstrapping calculation (1,000 replicates) was created to provide the phylogenetic trees that represent the clustering pattern among different species.

*Simulation method* - Due to the lack of establishment and maintenance of *S. gemmea* colonies for experimental infection, a simulation model of *L. martiniquensis* infection in *S. gemmea* sand flies was established for the validation of MG-LAMP, PCR-mkDNA, and PCR-ITS1 assays. The *S. gemmea* sand flies, collected from the bamboo plantations, were previously examined for *Leishmania* infection using PCR-mkDNA. The bodies of each uninfected *S. gemmea* were separated and consecutively used for the simulation models. The 2 x 10^5^ promastigotes/mL of *L. martiniquensis* were suspended in DNA extraction buffer. Each concentration of 10-fold serial dilutions from 10^4^ to 1 promastigote was made in 50 µL of DNA extraction buffer for each fly. The crude DNA was extracted from each fly, processed and stored as previously mentioned.

*Sensitivity, specificity, and field application* - To determine the sensitivity of MG-LAMP, PCR-mkDNA, and PCR-ITS1 to detect *L. martiniquensis* in sand flies, the 10-fold serial dilutions of *L. martiniquensis* (MHOM/TH/2011/PG) alone (10^4^ to 1 parasite in 75 µL; equivalent to 133 to 0.013 parasites/µL), and the crude extracts of *L. martiniquensis* with *S. gemmea* DNA (equivalent to 10^4^ to 1 parasite per sand fly) were used as the templates. The most sensitive method was defined as the method that could amplify the crude DNA extracted from the lowest number of promastigotes simulated in sand flies. To determine the specificity of each assay, cross amplification of other species of *Leishmania* and hemoparasites were also used as the template. The most specific method was defined as the method that could identify only *L. martiniquensis*. For field application, the most sensitive and specific amplification method was applied to detect *L. martiniquensis* parasites in 380 field captured female sand flies from the newly identified affected area of Tha Mae Lop Subdistrict, Mae Tha District, Lamphun Province (Chiewchanvit et al. 2015).

## RESULTS

*Sensitivity of MG-LAMP versus PCR-mkDNA* - MG-LAMP and PCR-mkDNA were successfully performed to amplify *L. martiniquensis* using *Leishmania* 18S rRNA-LAMP primers (Nzelu et al. 2014) and L.MC-1S/ L.MC-1R primers (Kato et al. 2007), respectively. Detection limit and cross-amplification of MG-LAMP and PCR-mkDNA assays were compared with PCR-ITS1 assay, which was reported as the most sensitive method for *L. siamensis* detection (Hitakarun et al. 2014). The sensitivities of these assays were assessed with the serial dilutions of *L. martiniquensis* (MHOM/TH/2011/PG) DNA alone (equivalent to 133 to 0.013 parasites/µL), and the crude extracts of *L. martiniquensis* mixed with *S. gemmea* DNA (equivalent to 10^4^ to 1 parasite per fly).

**Fig. 1:**
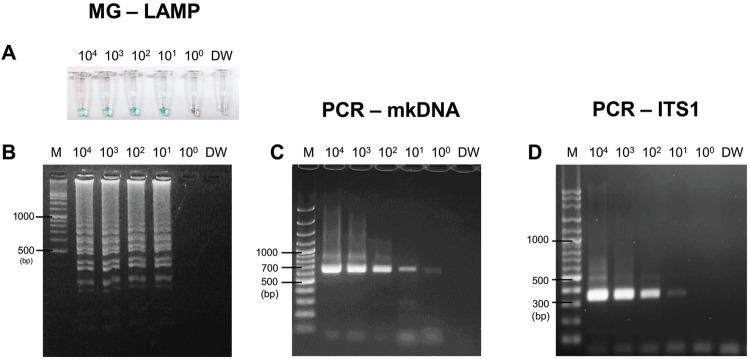
: sensitivity of malachite green-loop-mediated isothermal amplification (MG-LAMP), polymerase chain reaction of minicircle kinetoplast DNA gene (PCR-mkDNA), and PCR-ITS1 to detect *Leishmania martiniquensis*. Different concentrations of *L. martiniquensis* from 104 to 1 promastigote (equivalent to 133 to 0.013 parasites/µL) were used as the templates. (A) Visual detection of MG-LAMP; (B) agarose gel electrophoresis of MG-LAMP products; (C) agarose gel electrophoresis of PCR-mkDNA products; (D) agarose gel electrophoresis of PCR-ITS1 products. M: gene ruler; DW: distilled water (negative control).

**Fig. 2:**
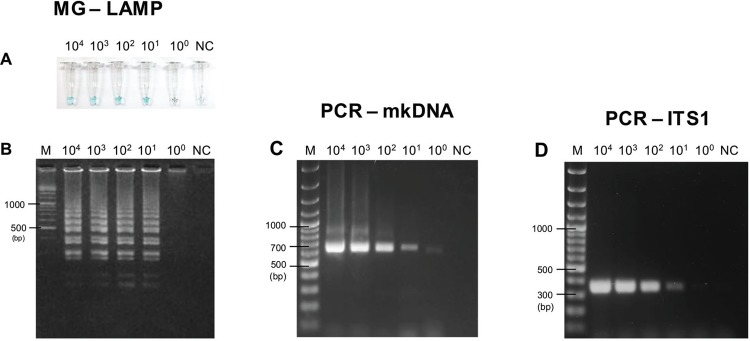
: sensitivity of malachite green-loop-mediated isothermal amplification (MG-LAMP), polymerase chain reaction of minicircle kinetoplast DNA gene (PCR-mkDNA), and PCR-ITS1 to detect *Leishmania martiniquensis* simulated in sand flies*.* Different concentrations of *L. martiniquensis* from 104 to 1 promastigote in *Sergentomyia gemmea* sand flies were used as the templates. (A) Visual detection of MG-LAMP; (B) agarose gel electrophoresis of MG-LAMP products; (C) agarose gel electrophoresis of PCR-mkDNA products; (D) agarose gel electrophoresis of PCR-ITS1 products. M: gene ruler; NC: *S. gemmea* alone (negative control).

PCR-mkDNA revealed a lowest detection limit at 0.013 parasites/µL or at least 1 promastigote of *L. martiniquensis* in one *S. gemmea* sample (Figs 1C, 2 C). Comparable to PCR-ITS1 assay, MG-LAMP was able to detect 0.13 parasites/µL (Fig. 1B) or 10 promastigotes of *L. martiniquensis* in one sand fly (Fig. 2B). No amplification was detected in the negative control using DW or sand fly alone. Positive results were visually discriminated when the sample turned light blue (Figs 1A, 2A), whereas the negative control turned from green to colorless. Gel electrophoresis also showed results in agreement with the colorimetric LAMP method using DNA intercalating malachite green dye (Fig. 1A versus 1B, and 2A versus 2B).

**Fig. 3:**
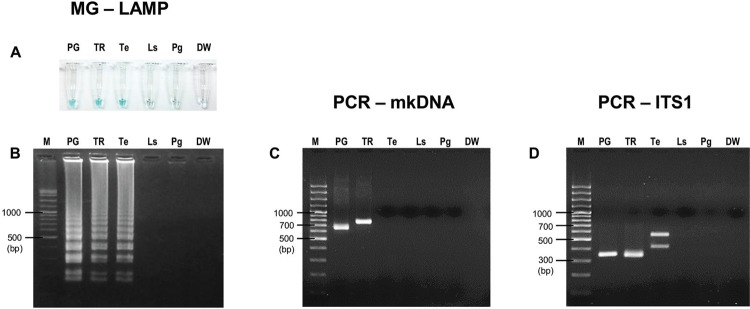
: specificity of malachite green-loop-mediated isothermal amplification (MG-LAMP), polymerase chain reaction of minicircle kinetoplast DNA gene (PCR-mkDNA), and PCR-ITS1 to detect *Leishmania martiniquensis*. (A) Visual detection of MG-LAMP; (B) agarose gel electrophoresis of MG-LAMP products; (C) agarose gel electrophoresis of PCR-mkDNA products; (D) agarose gel electrophoresis of PCR-ITS1 products. M: gene ruler; PG: *L. martiniquensis* (MHOM/TH/2011/PG); TR: *L. siamensis* (MHOM/TH/2010/TR); Te: *Trypanosoma evansi*; Ls: *Leucocytozoon sabrazesi*; Pg: *Plasmodium gallinaceum*; DW: distilled water (negative control).

**Fig. 4:**
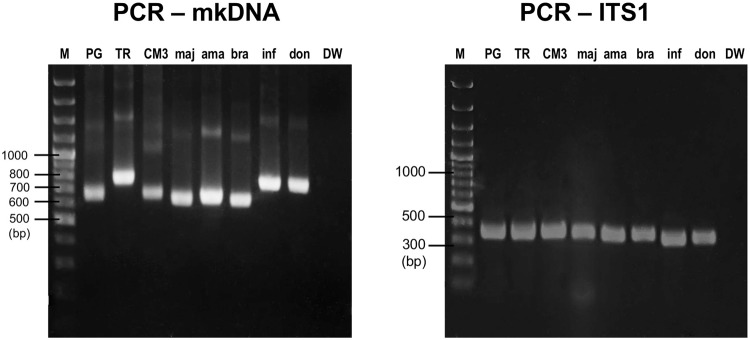
: detection of several species of *Leishmania* by polymerase chain reaction of minicircle kinetoplast DNA gene (PCR-mkDNA) and PCR-ITS1. Different amplicon’s sizes of PCR-mkDNA products of different species of *Leishmania* was observable. (A) Agarose gel electrophoresis of PCR-mkDNA products; (B) agarose gel electrophoresis of PCR-ITS1 products. M: gene ruler; PG: *Leishmania martiniquensis* (MHOM/TH/2011/PG); TR: *L. siamensis* (MHOM/TH/2010/TR); CM3: *L. martiniquensis* (MHOM/TH/2013/LSCM3); maj: *L. major*; ama: *L. amazonensis*; bra: *L. braziliensis*; inf: *L. infantum*; don: *L. donovani*; DW: distilled water (negative control).

*Specificity of MG-LAMP versus PCR-mkDNA* - DNA of *S. gemmea*, other *Leishmania* species, and some vector-borne protozoan parasites including *T. evansi*, *L. sabrazesi*, and *P. gallinaceum* were determined for cross-amplification of MG-LAMP, PCR-mkDNA, and PCR-ITS1 assays. All assays showed no cross-amplification with *S. gemmea* sand fly DNA. PCR-mkDNA assay was the most specific to amplify only *Leishmania* DNA, no cross-amplification with *T. evansi*, *L. sabrazesi*, and *P. gallinaceum* (Fig. 3C). Surprisingly, the L.MC-1S/ L.MC-1R primers, amplifying *Leishmania* mkDNA in this study, could discriminate between *L. martiniquensis* and *L. siamensis* with different PCR amplicon sizes, approximately 650 bp for *L. martiniquensis* and approximately 750 bp for *L. siamensis* (Fig. 3C)*.* On the other hand, MG-LAMP and PCR-ITS1 assays could amplify *T. evansi* but no reactivity was detected other avian haemosporozoan DNA samples (Fig. 3A-B, D). When amplified with other *Leishmania* species, PCR-ITS1 using primer L5.8S/LITSR could amplify *L. (L.) major*, *L. (L.) amazonensis*, *L. (V.) braziliensis*, *L. (L.) infantum*, and *L. (L.) donovani* with similar amplicon sizes, approximately 350 bp (Fig. 4B). PCR-mkDNA using primer L.MC-1S/ L.MC-1R was also able to amplify other *Leishmania* species. Similar results of PCR-mkDNA product at approximately 650 bp was observed when the assay amplified *L. martiniquensis* (MHOM/TH/2013/LSCM3) that was isolated from the patient from Mae Tha District. However, different amplicon sizes of PCR-mkDNA were found among *Leishmania* species, approximately 650 bp for *L. martiniquensis*, approximately 620 bp for *L. (L.) major*, *L. (L.) amazonensis*, and *L. (V.) braziliensis*, and longer than 700 bp for *L. siamensis*, *L. (L.) infantum*, and *L. (L.) donovani* (Fig. 4A).

**Fig. 5:**
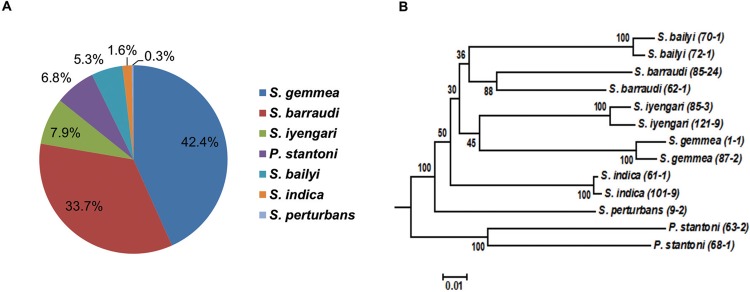
: phlebotomine sand flies fauna in the new affected area of Tha Mae Lop Subdistrict, Mae Tha District, Lamphun Province, Thailand. (A) Proportion of phlebotomine sand flies identifiable morphologically; (B) neighbor-joining tree based on Kimura 2 parameter genetic distances of mitochondrial COI sequences of phlebotomine sand flies. The bar scale represents 0.02% divergences. Bootstrap values are shown above or below branches. Specimen IDs (in parentheses).

*Molecular mass screening of sand flies from a new autochthonous leishmaniasis affected area* - The sensitivity and specificity results in a simulation model highlighted the potential of PCR-mkDNA to detect *L. martiniquensis* in sand flies. Therefore, PCR-mkDNA assay was consecutively applied to the mass screening of sand flies from the newly identified affected area of *L. martiniquensis* causing leishmaniasis in Tha Mae Lop Subdistrict, Mae Tha District, Lamphun Province (Chiewchanvit et al. 2015). The 380 captured female sand flies species were primarily identified microscopically and overall 7 species were morphologically identifiable, including *P. stantoni* (6.84%; 26/380), *S. gemmea* (42.37%; 161/380), *S. barraudi* (33.68%; 128/380), *S. iyengari* (7.89%; 30/380), *S. bailyi* (5.26%; 20/380), *S. indica* (1.58%; 6/380), and *S. perturbans* (0.26%; 1/380). Eight sand flies were morphological unidentifiable. To confirm the utility of the molecular mass screening procedure of sand flies; PCR and sequencing targeting the mitochondrial COI gene were conducted with the 13 identified sand flies with morphological differences. Phylogenetic analysis could discriminate seven groups of sand fly species, in agreement with morphological identification (Fig. 5). The 13 COI sequences of seven sand fly species in the present study are available in the DNA Data Bank of Japan (DDBJ) database under the accession numbers: *P. stantoni* (LC136898-LC136899); *S. gemmea* (LC136893-LC136894); *S. barraudi* (LC136902-LC136903); *S. iyengari* (LC136904-LC136905); *S. bailyi* (LC136900-LC136901); *S. indica* (LC136895-LC136896); and *S. perturbans* (LC136897). After the validity of mass screening was confirmed, the crude DNA extracts of 380 sand fly individuals were then used as a template for PCR-mkDNA to identify *L. martiniquensis*; however, the infection was undetected.

## DISCUSSION

The present study emphasised the high sensitivity and specificity of PCR-mkDNA to detect *L. martiniquensis*, at least one promastigote in a sand fly, and revealed the notable ability of PCR-mkDNA assay (L.MC-1S/ L.MC-1R primers) to discriminate *L. martiniquensis* from other viscerotropic *Leishmania* species, rendering this PCR-mkDNA assay as a promising tool for molecular mass screening of an individual sand fly for *L. martiniquensis* infection.

Due to various limitations in the microscopic detection of *Leishmania* in sand flies, several PCR-based techniques have been developed. The PCR-ITS1 (L5.8S/LITSR primers) has been reported as the most accurate method to detect *L. siamensis* (MHOM/TH/2010/TR), as low as 0.05 parasites/µL, and used as the reference assay to compare analytical and filed clinical sensitivity with PCR targeting the *18S rRNA*, *cyt* b, *heat shock protein* 70, *cysteine protease* B, spliced leader *mini-exon*, and *triose-phosphate isomerase* genes (Hitakarun et al. 2014). This assay has also been employed to detect *L. siamensis* and *L. martiniquensis* DNA within the sand fly pools captured from outbreak areas in southern Thailand (Kanjanopas et al. 2013, Chusri et al. 2014). However, the present study showed that PCR-ITS1 had 10-fold less sensitive than PCR-mkDNA and could not discriminate *L. martiniquensis* from other *Leishmania* species. The PCR-mkDNA may be an attractive molecular method to apply in the epidemiological study of *L. martiniquensis* infection in forthcoming outbreak.

Wide applicability of LAMP in the detection of parasitic protozoa such as *Babesia, Plasmodium*, *Trypanosome*, as well as *Leishmania* have been reported, due to its advantages, fast and simple amplification without the need of an expensive thermocycler. Recently, colorimetric malachite green based LAMP technique based on the *18S rRNA* gene was developed for the detection of *L. siamensis*, with the detection limit of at least 2.5 in clinical samples, such as whole blood and saliva (Sriworarat et al. 2015). The colorimetric LAMP protocol in the present study, however, showed a higher sensitivity to detect *L. martiniquensis* in a sand fly. Approximately 20-400 copies of the *ITS1* and *18S rRNA* gene in individual parasites have been described, although differing somewhat among *Leishmania* species (Inga et al. 1998).

The PCR-mkDNA using primers L.MC-1S/ L.MC-1R can detect at least one *L. (L.) major* existed in a sand fly sample (Kato et al. 2007). Along the similar line, this assay also showed the highest analytical sensitivity to detect *L. martiniquensis* even when only one promastigote existed in a sand fly sample, possibly due to the higher copy number of approximately 10,000 copies of the *mkDNA* gene in individual parasites (Simpson 1986). Regarding the specificity, this PCR-mkDNA protocol was the most specific to detect only *Leishmania* parasites, comparing to MG-LAMP and PCR-ITS1. The attractive feature of PCR-mkDNA is the ability to differentiate *L. martiniquensis* (MHOM/TH/2011/PG) clearly from other viscerotropic *Leishmania* species, including *L. siamensis*, *L. (L.) donovani*, and *L. (L.) infantum*, rendering the applicability of PCR-mkDNA for epidemiological study of VL caused by *L. martiniquensis* infection in the areas where several *Leishmania* species co-exist. Along similar lines, Kato et al. (2007) demonstrated that PCR-mkDNA worked on the other seven *Leishmania* species; *L. (L.) amazonensis*, *L. (L.) mexicana*, *L. (L.) major-like*, *L. (V.) panamensis*, *L. (V.) braziliensis*, *L. (L.) guyanensis* and *L. (L.) major*, although they have variations in their sequences. The differences in the size of amplified fragments among species may reflect the size of the dominant mkDNA in the strain because such DNA varies between 0.75 and 1 kbp in length (Brewster & Barker 2002). In addition, PCR of *kDNA* gene represent the most reliable tool to detect *L. infantum* naturally infection in *Lutzomyia longipalpis* in endemic areas of Brazil, comparing with *mini-exon* and *18S rRNA* genes (Freitas-Lidani et al. 2014). Lastly, the present study found that PCR-mkDNA showed no cross-amplification with *L. sabrazesi*, and *P. gallinaceum*, in which the potential vector of these avian hemosporidians are ceratopogonid midges. Seblova et al. (2015) suggested that *Culicoides soronensis* could be potential vectors of *L. enriettii*, relating to *L. martiniquensis* and *L. siamensis*. Validation of this PCR-mkDNA for detection of *Leishmania* in biting Culicoides midges should be further evaluated for research on ceratopogonid midges as the possible vector of *L. martiniquensis* and *L. siamensis* infection.

In Thailand, a few survey studies of the distribution of sand fly species and their habitats have been conducted. *Sergentomyia* fly was the most predominant genus found in the country. Until now, at least 26 species of sand fly have been reported in different provinces of Thailand, but only *S. barraudi* had been reported in Lumphun province (Polseela et al. 2016). The present study could provide more information of sand fly populations in Lumphun province, especially in the area where the affected patient resides. At least seven species of sand flies were identified, including *P. stantoni*, *S. gemmea*, *S. barraudi*, *S. iyengari*, *S. bailyi*, *S. perturbans*, and *S. indica,* of which *S. gemmea* and *S. barraudi* were the predominant species. Various studies demonstrated *Leishmania* DNA in *Sergentomyia* sand flies, e.g. *L. (L.) donovani* in *S. babu* in India (Mukherjee et al. 1997), *L. (L.) major* DNA in *S. minuta* in Portugal (Campino et al. 2013), as well as *L. siamensis* in *S. gemmea* (Kanjanopas et al. 2013), and *L. martiniquensis* in *S. gemmea* and *S. barraudi* in Thailand (Chusri et al. 2014). Moreover, *L. (L.) major* was also isolated from *S. garnhami* and successfully cultured in NNN medium (Mutinga et al. 1994). In this study, *Leishmania* DNA was not detected, probably due to the very low infection rate (0.01-1%) among sand fly populations even in endemic areas (Kato et al. 2016). Further surveillance of larger populations using the present mass screening approach will provide more information about sand flies in each endemic area. The abundance of *S. gemmea* and *S. barraudi* in the local environment of the affected patient may raise awareness of public health concerns for prevention and control of leishmaniasis among policy- and decision-makers, physicians and the general public. Further surveillance of larger populations using mass screening will provide more information.

In conclusion, the present study highlighted the potential of PCR-mkDNA method as a promising tool to detect *L. martiniquensis* in sand flies due to its high sensitivity and specificity. Above all, PCR-mkDNA has the valuable ability to discriminate between *L. martiniquensis* and other viscerotropic *Leishmania* species; *L. siamensis*, *L. (L.) donovani*, and *L. (L.) infantum*, which may encourage researchers to adopt this approach for epidemiological studies of VL in such areas where many *Leishmania* species are circulating. Identifying the potential vector for *L. martiniquensis* still remains an urgent needed. The molecular mass screening of individual sand fly for *Leishmania* infection by PCR-mkDNA is applicable to provide informative data on the vector and vector-*Leishmania* relationship in outbreak areas where several *Leishmania* and sand fly species co-exist and the species of potential vectors remain unknown.
