# Supplementary material for: Comparison of LAMP and PCR for molecular mass screening of sand flies for Leishmania martiniquensis infection
Source: Mem Inst Oswaldo Cruz. 2017 Feb;112(2):100–7. doi: 10.1590/0074-02760160254 (PMC5293119; doi:10.1590/0074-02760160254)
Supplement: Supplementary file 1 [file 0074-0276-mioc-112-2-0100-suppl01.pdf]

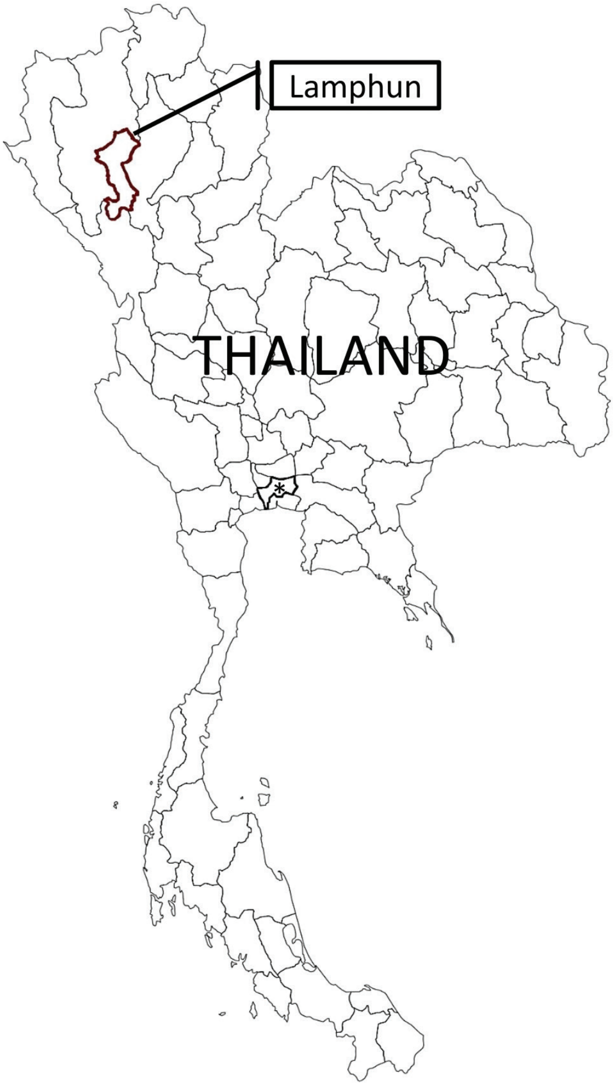

Map of the new affected areas of Lamphun Province, Thailand. Bangkok (*) as the capital city (Adapted from a map available from: http://simplemaps.com/resources/svg-th).
